# Relationship Between Cytokine Gene Polymorphisms and Risk of Postoperative Pneumonia with Esophageal Cancer

**DOI:** 10.1007/s11605-014-2531-3

**Published:** 2014-05-08

**Authors:** Kazuhiko Sakamoto, Masaaki Oka, Shigehumi Yoshino, Shoichi Hazama, Shigeru Takeda, Kiyoshi Yoshimura, Naoko Okayama, Yuji Hinoda

**Affiliations:** 1 Department of Digestive Surgery and Surgical Oncology, Yamaguchi University Graduate School of Medicine, 1-1-1 Minami-Kogushi, Ube, Yamaguchi 755-8505 Japan; 2 Division of Laboratory, Yamaguchi University Hospital, Yamaguchi, Japan; 3 Department of Oncology and Laboratory Medicine, Yamaguchi University Graduate School of Medicine, Yamaguchi, Japan

**Keywords:** IL-10 polymorphism, Postoperative pneumonia, Esophagectomy

## Abstract

**Background:**

We retrospectively evaluated the relationship between cytokine gene polymorphisms and development of postoperative pneumonia after esophagectomy.

**Methods:**

In 120 patients who underwent esophagectomy, serum samples were obtained to measure levels of serum interleukin (IL)-6 and IL-10 at four time points (preoperatively, postoperative day (POD)0, POD1, and POD3). DNA extracted from peripheral blood in all patients was analyzed to determine polymorphisms of cytokines such as tumor necrosis factor-α -1031 T/C, IL-1β -511C/T, IL-6 -634C/G, and IL-10 -819 T/C.

**Results:**

Postoperative pneumonia arose in 34 patients (28.3 %). Perioperative serum IL-10 levels were significantly higher for IL-10 -819 C/T + C/C genotypes than for T/T genotypes (POD0 16.7 ± 2.84 vs. 8.54 ± 0.87 pg/ml, *p* = 0.0002; POD1 14.0 ± 2.64 vs. 8.8 ± 0.87 pg/ml, *p* = 0.0143; POD3 8.9 ± 2.67 vs. 4.4 ± 0.52 pg/ml, *p* = 0.0076). The frequency of the IL-10 -819 T/T genotype was significantly higher in patients with postoperative pneumonia than in patients without pneumonia (*p* = 0.0323). Multivariate analysis of factors such as sex, smoking, length of operation, field of lymph node dissection, and IL-10 polymorphism identified IL-10 polymorphism as independent predictor of postoperative pneumonia.

**Conclusions:**

Patients with IL-10 -819 T/T genotype may be at high risk for postoperative pneumonia after esophagectomy.

## Introduction

Surgery for esophageal cancer is one of the most invasive gastrointestinal surgeries,^1^, ^2^ and perioperative mortality rates remain in the range of 3–10 %.^3^^–^ 5 In the case of esophageal cancer, marked elevations in cytokine levels are observed perioperatively, inducing a systemic inflammatory response syndrome (SIRS).^6^, ^7^ Such hypercytokinemia produces excessive stress and may trigger postoperative complications.^8^ The lungs is one of the main organs of neutrophil sequestration under conditions of SIRS.^9^^–^ 12 Abe et al. reported that transthoracic esophagectomy causes an increase in interleukin (IL)-6 production from bronchial and alveolar epithelial cells lining the airway, and local response of lung tissue may be one source of increased serum IL-6 after esophagectomy in humans.^13^ The clinical effects of an immune response are orchestrated by numerous pro-inflammatory, inflammatory, and anti-inflammatory cytokines. Tumor necrosis factor (TNF)-α is a major mediator and amplifier of immune responses to infectious challenges. Azim et al. recently reported that the TNF-α -308 polymorphism contributes to infectious complications after esophagectomy.^14^ Moreover, associations between infection and genetic polymorphisms have been reported for other cytokines, including IL-1β, IL-6, and IL-10.^15^^–^ 18 These findings suggest that patient genotype may influence susceptibility to postoperative infection.

We have a considerable interest in whether factors such as these polymorphisms might affect the postoperative infection, particularly in terms of the effects on postoperative pneumonia. We selected TNF-α, IL-1β, IL-6, and IL-10 as representative pro-inflammatory, inflammatory, and anti-inflammatory cytokines and investigated the gene promoter polymorphisms of TNF-α -1031 T > C (rs1799964), IL-1β -511 T > C (rs3087258), IL-6 -634G > C (rs1800796), and IL-10 -819 T > C (rs1800871); all of which have been reported to influence cytokine production.^18^^–^ 22 The purpose of this study was to assess whether cytokine promoter gene polymorphisms are associated with the following: (1) perioperative cytokine production and (2) postoperative pneumonia following esophagectomy.

## Methods

### Patients

This was a retrospective cohort study that included patients treated for esophageal cancer between 1997 and 2011. Participants comprised 120 consecutive Japanese patients (105 men and 15 women) with thoracic esophageal cancer who underwent curative esophagectomy and reconstruction with gastric mobilization via a posterior mediastinal or retrosternal route by right posterolateral thoracotomy and laparotomy consecutively in the Department of Digestive Surgery and Surgical Oncology at Yamaguchi University Graduate School of Medicine. These patients underwent dissection of two (mediastinal and abdominal) or three (bilateral neck, mediastinal, and abdominal) lymph node fields. Patients who received neoadjuvant treatment or underwent reconstruction with small intestine or colon mobilization were excluded. We introduced thoracoscopic procedures in a prone position from 2008 and excluded these procedures from the present study. In all cases, diagnoses of esophageal squamous cell cancer were confirmed preoperatively on the basis of histopathological reports. The clinicopathological definition of thoracic esophageal cancer was based on the International Union against Cancer tumor-node-metastasis (TNM) classification of malignant tumors (6th edition). Written informed consent was obtained from all study patients. The study protocol was approved by the Institutional Review Board for the Use of Human Subjects at Yamaguchi University Graduate School of Medicine.

### Perioperative Management

We performed perioperative management as follows. At the first visit to our hospital, all patients were instructed to quit smoking and respiratory rehabilitation was started at the same time. As prophylactic antibiotics, patients received cefazolin 1 g by intravenous infusion for 30 min before the operation. An additional dose was administered if the operation was prolonged beyond 3 h. Patients received the same antibiotics plus further treatment at 12-h intervals, for a total of 5 days. For nutritional support, enteral nutrition was provided via jejunostomy from 6 h postoperatively, and the dose was increased step-by-step to 2,000 kcal/day up to postoperative day (POD) 7. All patients were admitted to the intensive care unit and placed on prophylactic mechanical ventilation. The timing of extubation was decided according to the results of bronchoscopy, blood gas analysis, and chest radiography. The criteria for extubation were as follows: (1) arterial oxygen pressure >100 mmHg with inspired fraction of oxygen (FiO_2_) 0.4 and (2) absence of atelectasis on bronchoscopic view or radiography.

### Postoperative Complications

Postoperative complications were defined as follows. Postoperative pneumonia was diagnosed by the presence of pyrexia >38 °C within 2 weeks postoperatively and either positive sputum cultures or clear clinical or radiological evidence of consolidation. Anastomotic leakage was diagnosed by gastrography and clinical features. Postoperative wound infection was defined as a systemic response to the presence of an infectious agent supported by clinical and laboratory evidence, where laboratory evidence means positive culture results. Cardiac complications including arrhythmia, angina, or heart failure were diagnosed by a cardiologist.

### Serum Cytokines

Serum samples were obtained at four time points (preoperatively, end of the operation (POD0), POD1, and POD3) and stored at −80 °C until use. Serum IL-6 and IL-10 levels were measured using commercially available enzyme-linked immunosorbent assay kits (BioSource International, Camarillo, CA). Samples were prepared and tested in duplicate according to the protocol recommended by the manufacturer.

### DNA Specimens and Genotyping

For DNA analysis, 7 ml of peripheral blood was obtained from all patients. DNA was isolated by a conventional NaI method and stored at 4 °C.^23^ All polymorphisms were identified with the tetra-primer amplification refractory mutation system-polymerase chain reaction (ARMS-PCR), and details of primers and PCR conditions have been described in the literature.^24^^–^ 26

### Statistical Analysis

Data are expressed as mean values±standard error and were analyzed using the Mann-Whitney *U* test. Categorical data were analyzed using the χ^2^ test or Fisher’s exact test. Differences in genotype frequency were analyzed using the χ^2^ test or Fisher’s exact test of independence, with which, each of the genotype frequencies was evaluated to determine whether it was consistent with expected Hardy-Weinberg proportions. Because homozygotes for the rare allele were too few to perform a 2 × 3 χ^2^ test or Fisher’s exact test, homozygotes of the dominant alleles and variant carriers were compared by 2 × 2 test. Variables with a *p* < 0.2 in the univariate analysis that were potentially predictive of postoperative pneumonia were then entered into the multivariate logistic regression model. Odds ratio (OR) and 95 % confidence interval (CI) were also calculated. A value of *p* < 0.05 was considered statistically significant. All analyses were performed using StatView version 5.0 statistical software (SAS Institute, Cary, NC).

## Results

In all 120 patients studied, the frequency of postoperative pneumonia was 28.3 % (34 of 120 patients). In terms of other complications, 14 patients (11.7 %) developed wound infection, 8 patients (6.7 %) developed anastomotic leakage, and 25 patients (20.8 %) developed cardiac complications. Perioperative patient- and tumor-related factors and other complications were compared between groups with and without postoperative pneumonia (Table 1). The proportion of males was significantly higher among patients with pneumonia (97.1 %) than among patients without pneumonia (82.6 %; *p* = 0.0384). The proportions of smoking, 3-field lymph node dissection, and the length of operation tended to be higher among patients with pneumonia than among patients without pneumonia (*p* = 0.147, *p* = 0.102, and *p* = 0.0679, respectively).

**Table 1:** Patient characteristics in the two groups

	Pneumonia + (*n* = 34)	Pneumonia − (*n* = 86)	*p* value
Age	63.7 ± 8.9	63.0 ± 9.2	0.337
Gender			
Male	33	71	
Female	01	15	0.0384
Body mass index (kg/m^2^)	21.0 ± 3.0	21.1 ± 3.0	0.954
Smoking			
Yes	32	71	
No	02	15	0.147
FEV1.0 %	73.2 ± 12.2	73.1 ± 8.8	0.592
Preoperative CRP (mg/dl)	0.82 ± 2.4	0.32 ± 0.47	0.765
Tumor locations			
Upper	04	09	
Middle	21	44	
Lower	09	33	0.465
UICC stage (pathological)			
I and II	21	50	
III and IV	13	36	0.716
Length of operation (min)	463 ± 100	433 ± 99	0.0679
Blood loss (g)	820 ± 913	548 ± 267	0.283
Field of lymph node dissection			
2 (mediastinum and abdomen)	22	68	
3 (bilateral neck, mediastinum, and abdomen)	12	18	0.102
Wound infection			
Yes	5	09	
No	29	77	0.536
Anastomotic leakage			
Yes	03	05	
No	31	81	0.686
Cardiac complications			
Yes	09	16	
No	25	70	0.339

Data are presented as mean+standard error or absolute numbers

*FEV* forced expiratory volume, *CRP* C-reactive protein, *UICC* International Union Against Cancer

**Fig 1:**
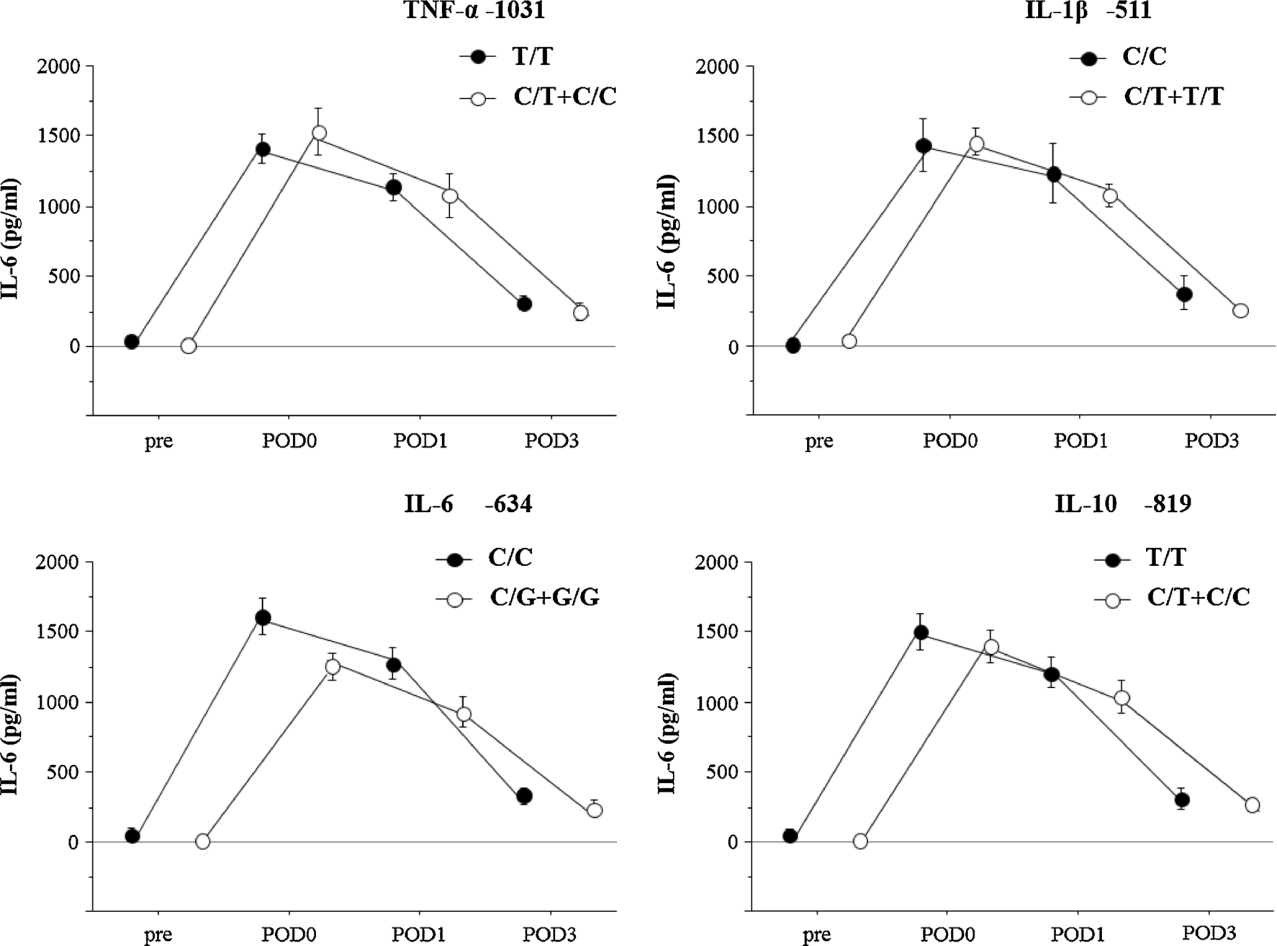
Pre- and postoperative serum levels of interleukin (IL)-6. The relationship between polymorphism of the cytokine genes (tumor necrosis factor (TNF)-α -1031 T/C, IL-1β -511C/T, IL-6 -634C/G, IL-10 -819 T/C) and IL-6 level in 120 patients after esophagectomy. *Solid circles* indicate wild-type polymorphism, *open circles* indicate variant-type polymorphism. Values are expressed as mean±standard error. Statistically significant according to the Mann-Whitney *U* test. *IL* interleukin, *TNF* tumor necrosis factor, *POD* postoperative day

**Fig 2:**
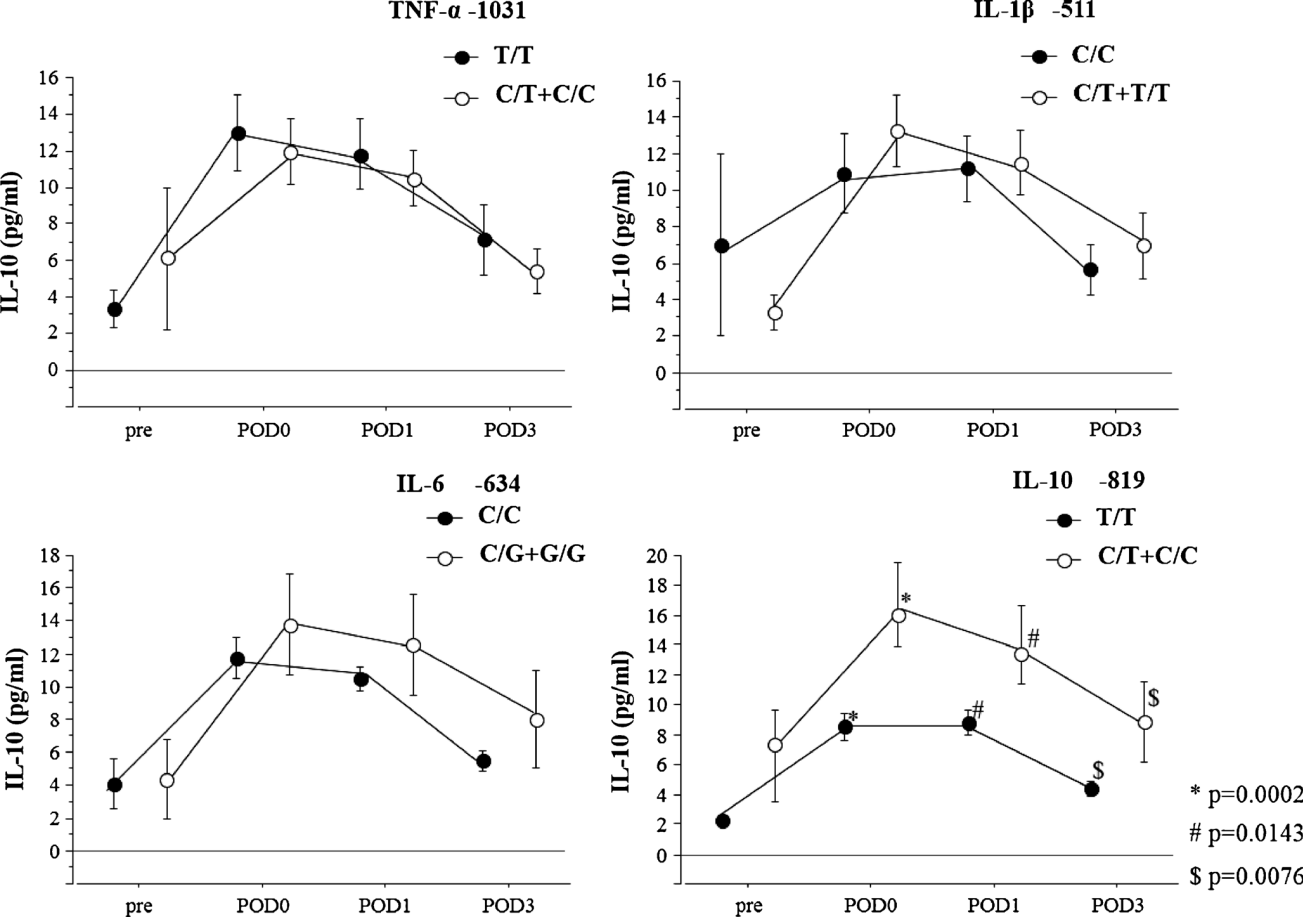
Pre- and postoperative serum levels of interleukin (IL)-10. The relationship between polymorphism of cytokine genes (tumor necrosis factor (TNF)-α -1031 T/C, IL-1β -511C/T, IL-6 -634C/G, IL-10 -819 T/C) and IL-10 level in 120 patients after esophagectomy. *Solid circles* indicate wild-type polymorphism, *open circles* indicate variant-type polymorphism. Values are expressed as mean±standard error. Statistically significant according to the Mann-Whitney *U* test. ^*^ *p* = 0.0002; ^#^ *p* = 0.0143; ^$^ *p* = 0.0076. *IL* interleukin, *TNF* tumor necrosis factor, *POD* postoperative day

No relationship was seen between perioperative serum IL-6 levels and polymorphisms (Fig. 1). Perioperative serum IL-10 levels were significantly higher for IL-10 -819 C/T+C/C genotypes than for T/T genotypes (POD0 16.7 ± 2.84 vs. 8.54 ± 0.87 pg/ml, *p* = 0.0002; POD1 14.0 ± 2.64 vs. 8.8 ± 0.87 pg/ml, *p* = 0.0143; POD3 8.9 ± 2.69 vs. 4.4 ± 0.52 pg/ml, *p* = 0.0076) (Fig. 2).

Among the cytokine promoter gene polymorphisms analyzed, a significant association with pneumonia was found for IL-10 -819 T/C. The frequency of the IL-10 -819 T/T genotype was significantly higher in patients with pneumonia than in those without pneumonia (*p* = 0.0323) (Table 2).

**Table 2:** Genotype distribution in the postoperative pneumonia

Cytokine polymorphism		Pneumonia + (*n* = 34)	Pneumonia − (*n* = 86)	*p* value
TNF-α -1031	T/T	21	62	0.27
	T/C + C/C	13	24	
IL-1β -511	C/C	08	26	0.463
	C/T+T/T	26	60	
IL-6 -634	G/G	20	47	0.678
	G/C+C/C	14	39	
IL-10 -819	T/T	22	37	0.0323
	T/C+C/C	12	49	

*TNF* tumor necrosis factor, *IL* interleukin

Although no relationship was seen between postoperative serum IL-10 levels and Il-10 polymorphisms in patients with pneumonia, these levels were significantly higher for IL-10 -819 T/C + C/C genotypes than for T/T genotypes in patients without pneumonia (Table 3).

**Table 3:** Trend in serum IL-10 levels according to IL-10 -819 polymorphism with and without pneumonia

	Pneumonia ± (*n* = 34)		*p* value	Pneumonia − (*n* = 86)		*p* value
	T/T (*n* = 22)	T/C±C/C (*n* = 12)		T/T (*n* = 37)	T/C±C/C (*n* = 49)	
Serum IL-10 level (pg/ml)						
POD0	9.5 ± 1.6	17.4 ± 5.3	0.65	8.0 ± 1.0	16.5 ± 3.3	<0.01
POD1	10.1 ± 1.8	10.9 ± 4.0	0.62	8.0 ± 0.9	14.8 ± 3.2	<0.01
POD3	5.3 ± 0.9	6.2 ± 3.0	0.77	3.8 ± 0.6	9.6 ± 3.3	<0.01

Data are presented as mean±standard error

*IL* interleukin, *POD* postoperative day

Clinicopathological variables, including sex (male vs. female), smoking (yes vs. no), length of operation, field of lymph node dissection (three vs. two), and IL-10 -819 polymorphism (T/T vs. C/T + C/C) were entered into a multivariate logistic regression model to identify factors influencing pneumonia (Table 4). Only IL-10 -819 polymorphism was significantly associated with pneumonia (*p* = 0.0334, OR = 2.68, 95 % CI = 1.08–6.67).

**Table 4:** Logistic regression predicting development of postoperative pneumonia

	OR	95 % CI	*p* value
Gender (male vs. female)	4.64	0.491–43.9	0.18
Smoking (yes vs. no)	1.35	0.235–7.72	0.738
Length of operation (min)	1.002	0.998–1.007	0.276
Field of lymph node dissection (3 vs. 2)	1.43	0.516–3.97	0.491
IL-10 Genotype (T/T vs. C/T+C/C)	2.68	1.08–6.67	0.0334

*OR* odds ratios, *CI* confidence interval, *IL* interleukin

## Discussion

This study revealed that IL-10 -819 polymorphism is associated with postoperative pneumonia in Japanese patients with esophageal cancer. The IL-10 -819 TT genotype is related to lower perioperative production of serum IL-10 level. No associations with any other cytokine polymorphism for TNF-α, IL-1β, or IL-6 were evident. Presence of the IL-10 -819 TT genotype seems to confer lower IL-10 release in response to surgical stress and an increased risk of postoperative pneumonia.

Under highly inflammatory conditions such as following esophagectomy, a genotype associated with high anti-inflammatory response may be protective against the development of postoperative respiratory inflammation. IL-10 is a potent endogenous anti-inflammatory cytokine that decreases lung inflammation, partly on the basis of TNF-α and IL-1β.^27^ Alveolar macrophages produce significant amounts of IL-10.^28^, ^29^ Morita reported that IL-10 production after rat thoracotomy was higher from the collapsed lung than from the uncollapsed lung, possibly reflecting suppression of IL-6 production from the collapsed lung.^30^ These findings are supported by the clinical observation that decreased IL-10 concentration in bronchoalveolar lavage fluid is associated with worse outcomes in patients with the acute respiratory distress syndrome.^31^ IL-10 therapy increased survival both in a rabbit *Pseudomonas aeruginosa* pneumonia model and in a murine pneumococcal pneumonia model.^32^ Those results indicate that IL-10 is a key regulator of the degree of inflammation in the setting of acute lung infection or inflammation.

The current finding that the high IL-10-producing -819C allele is protective against postoperative respiratory inflammation following esophagectomy is consistent with the role of intense inflammation. Lowe et al. reported that the IL-10 -592 CC polymorphism was associated with higher IL-10 release under lipopolysaccharide stimulation and lower mortality in critically ill patients.^20^ Similar frequencies were obtained for -819 T/C and -592 A/C in all types of participants, indicating complete linkage.^33^ Those findings are consistent with our own results. However, in critically ill patients with sepsis, the mortality rate was significantly higher in patients with the -592 CC genotype than in those with the -592 AA genotype.^33^ The overall balance between pro- and anti-inflammatory responses is important in response to injury or surgery. In patients with sepsis, a genotype associated with high IL-10 production may lead to greater immunosuppression, resulting in more severe disease burden and outcomes. In contrast, the present findings were obtained following esophagectomy. In such cases, a genotype associated with high IL-10 may have beneficial modulating effects. The notion that a high IL-10-producing -819 C allele may not be universally detrimental appears reasonable.

Azim et al. reported that not IL-10 -819 T/C but TNF-α-308G/A polymorphism contributes to infectious complications after esophagectomy.^14^ That result differs from the findings of the present study. However, the frequency of the -308 A allele for TNF-α is much lower in Japanese populations (1.1–1.3 %) than in Caucasians (10–18 %).^33^^–^ 36 Furthermore, the frequency of the -819 C allele of IL-10 in Japanese (30.5–33.6 %) was lower than in Caucasians (79–82 %).^20^, ^33^, ^37^ These results suggest that considering the influence of ethnic differences in gene polymorphism may be important. Further studies on gene polymorphisms in different ethnicities are needed to allow tailoring of specific therapies. Identifying IL-10 polymorphisms in patients prior to esophagectomy may influence management decisions where controversy currently exists, such as the use of other therapeutic strategies. For example, whether steroids should be used to treat patients perioperatively remains unresolved.^38^ Some patients may benefit, while others may not, depending on individual genotypes. Genotyping this IL-10 polymorphism in patients following esophagectomy may allow better risk stratification and tailoring of specific therapies to different risk groups.
